# The relationship between occupational stress, mental health and work ability of coal chemical workers in Xinjiang

**DOI:** 10.3389/fpsyt.2022.903534

**Published:** 2022-08-18

**Authors:** Xiaoting Yi, Jun Yang, Xiaoyan Gao, Fuye Li

**Affiliations:** Department of Public Health, Xinjiang Medical University, Ürümqi, China

**Keywords:** occupational stress, mental health, work ability, coal chemical worker, structural equation

## Abstract

**Objective:**

To understand the current working ability of coal chemical workers in Xinjiang, and analyze the impact of occupational stress, mental disorders, and their interaction on work ability. To provide a scientific basis for improving the working ability and quality of life of coal chemical workers.

**Methods:**

In this study, a stratified random sampling method was used to conduct a questionnaire survey of 1,400 workers in six coal mining enterprises from June to December 2019. The Effort-Reward Imbalance Questionnaire (ERI), the Symptoms Checklist 90 (SCL-90), and the Work Ability Index Scale (WAI) were used to evaluate the level of occupational stress, mental disorders, and the ability to work as coal chemical workers.

**Results:**

The results showed that WAI scores had statistically significant differences between gender, age, length of service, shift, education, marital status, and monthly income (*P* < 0.05). The ability to work and its dimensions were inversely correlated with occupational stress, and mental disorders (*P* < 0.001). Occupational stress and mental disorders are risk factors affecting the ability to work. Workers with high occupational stress^*^ mental disorders (OR = 10.666, 95% CI: 6.443–17.658) are 10.666 times more likely to be at risk for developing poor work ability than low occupational stress^*^ no mental disorders. Structural equation models show that occupational stress and mental health conditions had a direct impact on work ability, and mental health conditions were the mediators of the relationship between occupational stress and work ability.

**Conclusion:**

Occupational stress, mental disorders, and their interaction are risk factors affecting the ability of coal chemical workers to work. Occupational stress can indirectly affect the ability to work through mental health conditions.

## Graphical Abstract

It shows that the occupational stress of the coal chemical industry can directly act on work ability, and can also indirectly act on work ability through mental health status as an intermediary variable. 
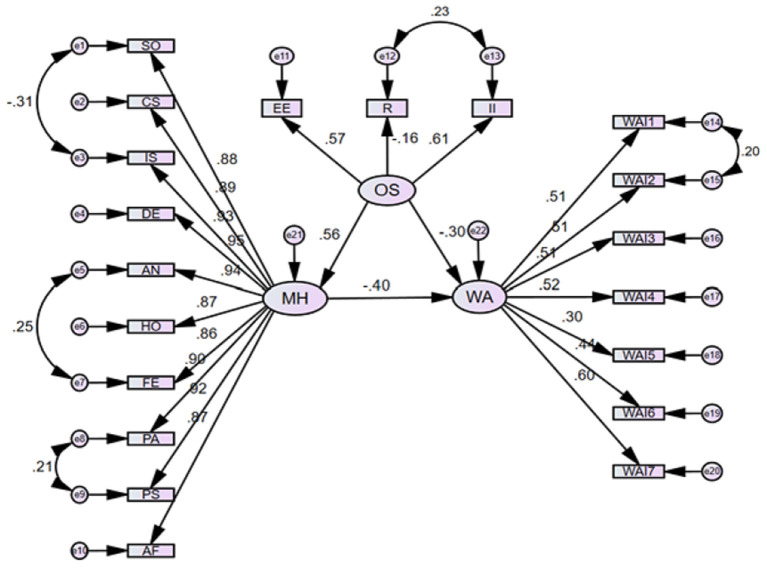

## Background

Occupational stress is a psychological and physiological reaction that occurs when the individual's work ability and work requirements are not equal (1). In general, the effects of occupational stress on individuals are manifested early in psychological aspects, such as psychological symptoms such as anxiety and hostility (2, 3). When an individual is in a stressful environment for a long time and cannot cope, the long-term exposure to stress may produce typical characteristics, such as ineffective behavior and excessive reactions, which increase the health risk (4, 5). As a psychological factor, although occupational stress cannot cause specific occupational diseases, occupational stress can act on the body for a long time, so that the body is in a bad state of health, thereby causing damage to the body, mind, and spirit in a non-specific way, thus affecting its ability to work (6). Studies have shown that different occupational groups have different levels of stress at work, with teachers (7), medical workers (8), oil workers (9), and coal miners (10) being high-risk occupational groups for occupational stress. Studies by scholars abroad have shown that when the body is in a state of high tension for a long time, the body might develop coronary heart disease, primary hypertension, and other physical diseases, and occupational stress is also inseparable from the incidence of musculoskeletal diseases (11–13).

In addition to causing damage to physical health, occupational stress also has an impact on mental health, including cognitive ability and emotional states (emotional state, intellectual activity, level of consciousness, and other psychological conditions). The mental effects of occupational stress can range from mild subjective symptoms to significant mental abnormalities and even psychosis, with common symptoms, such as irritability, decreased job satisfaction, depression, nervousness, and anxiety (14–16). Mental disorders accounted for 2295.8 of age-standardized DALYs per 100,000 people and substance-use disorders accounted for 399.9 age-standardized DALYs per 100,000 in 2019, with 1.8% and 20.1% increases, respectively, compared with 1990 (17). The number of incident cases of depression worldwide increased from 172 million in 1990 to 25,8 million in 2017, representing an increase of 49.86% (18). It is expected that in the coming years, mental illness may become the most expensive disease of health resources in China (19). Greater than one-fourth of the world's occupational population will be engaged in professional activities, and their productivity will be plagued by mental health problems. In some professional groups with a high degree of tension, psychological problems will occur when psychological pressure accumulates to a certain extent and there is no suitable way to decompress (20).

Due to the long-term existence of stress factors that lead to individual occurrences, the psychological and physical health levels of the occupational population decline, showing a series of negative physiological, psychological, and behavioral effects that have a negative impact on the working ability of workers (21). This situation will cause economic losses to enterprises and countries. The impact of stressful work efficiency has become an important occupational health problem at home and abroad. Studies have found that mental health status is related to work efficiency, and a good mental state can help mobilize the enthusiasm of employees (22). Aqeel et al. (23) results show that employee job stress is a positive and significant predictor of job separation intentions. In addition, people affected by mental health problems are also vulnerable in the management of family and social events, with a sense of frustration at work, absenteeism at work, and high unemployment rates (24).

Coal chemical workers operate in a special nature, and their workplaces are often accompanied by various types of occupational hazards, such as high temperatures, coal soot, radiation, and toxic and harmful gases (25). Coal chemical workers are mostly manual workers, some types of work are highly dangerous and mentally stressful, the workplace unsafe conditions are high, and they are prone to work accidents (26). At present, the coal chemical workers have a low level of education, worse living habits, greater emotional fluctuations, weak self-regulation ability, and low attention to mental health. In addition, coal chemical workers also face tense life pressure, work instability, and other factors, which makes them have great mental and psychological pressure. Studies have found that the population of psychological stress-induced diseases is increasingly large, resulting in a gradual decline or even loss of employees' ability to work (27). At present, the research related to occupational stress and work ability is mainly based on medical, educational, and petroleum occupational groups, and there are fewer studies on the current situation and influencing factors of coal chemical workers on occupational stress, mental health and work ability. Numerous studies have established a correlation between occupational stress, mental health, and work capacity (28, 29). However, the structural relationship between mental health, occupational stress, and ability to work has not been explored in depth. In this study, the occupational stress, mental health, and working ability among employees of Xinjiang coal chemical workers are proposed as the main analysis content and the relationship between the three. Structural equation modeling analysis of the mediating effect of mental health in the relationship between occupational stress and work ability is required.

## Measures

### Subjects

**Figure 1:**
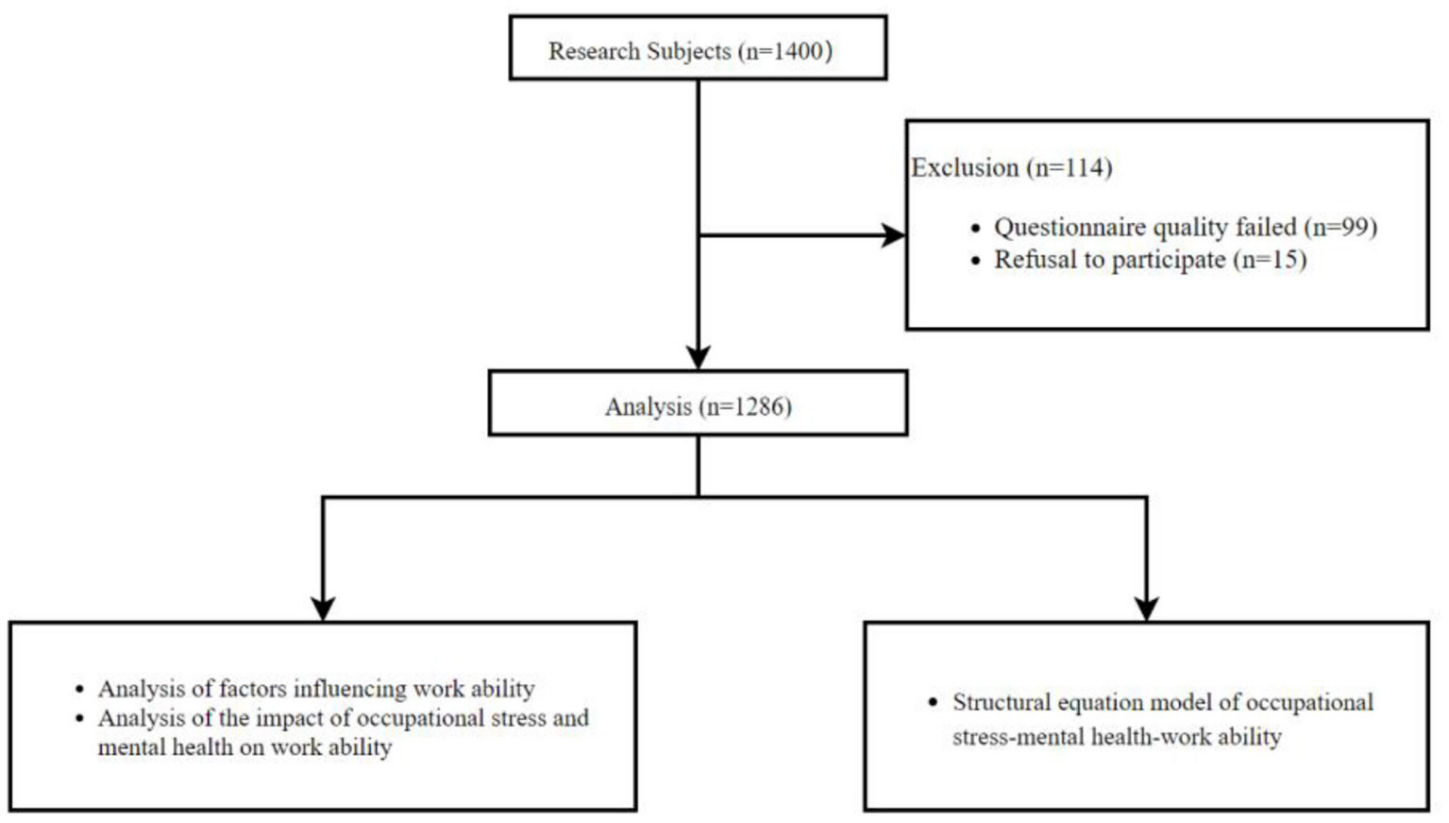
The study flow diagram.

Xinjiang coal chemical workers as the target group of this study. This study used a stratified cluster random sampling method from June 2019 to December 2019. According to the annual output, Xinjiang coal chemical enterprises are divided into three production levels: high (annual output ≥ 1.2 million tons), medium (annual output < 1.2 million tons), and low (annual output < 450,000 tons), and 2 coal chemical enterprises are randomly selected for investigation at each level. A total of 1,400 coal chemical workers were surveyed in this study, and a total of 1,286 questionnaires were recovered by excluding those whose questionnaire quality was unqualified, with a recovery rate of 91.86%. Inclusion criteria: (1) Age ≥18 years; (2) length of service ≥ 1 year; (3) Workers provide informed consent and voluntarily participate in the study after investigators have informed the purpose and significance of the study. The research proposal was approved by the Ethics Committee of Xinjiang Medical University (the ethical NO.20170214-174). Prior to the start of the survey, all respondents voluntarily provided written informed consent forms. The flowchart is shown in Figure 1.

### Occupational stress measurement

The Effort-Reward Imbalance Q*uestionnair*e (ERI) was used to measure the occupational stress of survey respondents. This scale was proposed by Siegrist (30) and adapted by Li et al. (31) into the Chinese edition of the ERI questionnaire, which has good reliability and validity (32). The questionnaire combined the environmental (paying, reward dimensions) and individual factors (intrinsic input dimensions) to interpret the psychological tension of the respondents. The questionnaire included 23 items in terms of external effort (6 items), reward (11 items), and intrinsic input (6 items). ERI ratio was used as the outcome indicator: ERI = external effort / (return ^*^ C), where constant C = 6 / 11. An ERI ≤ 1 represents a low pay-high return, which indicates low-level occupational stress; an ERI > 1 represents a high pay-low return, indicating high-level occupational stress (33).

### Determination of mental health status

The mental health of respondents was measured using the Symptoms Checklist 90 (SCL-90) and showed high reliability and validity among all groups (34, 35). This scale was based on the Symptoms Checklist 90 (Hopkins Symptom Checklist, 1973) compiled by Derogatis (36), and mental symptoms were assessed using a self-rated scale which consisted of a total of 90 items. The subscale contained nine factors, which included somatization, compulsive symptoms, interpersonal sensitivity, depression, anxiety, hostility, fear, paranoia, psychosis, as well as another factor. Responses were measured using a scale ranging from 1 to 5, such that 1 = none, 2 = mild, 3 = moderate, 4 = severe, and 5 = severe. The total score was obtained from the sum of the scores for all of the items, and the total score of each factor item was obtained by the sum of the factors. A total score of more than 160 points or a factor score of more than 2 points was taken to indicate a psychological disorder (37). With respect to the measurement results, the present study referred to Jin et al. (38) research, which obtained the data of 1,388 normal people from 13 regions in China, and this was taken as the norm.

### Determination of working ability

The use of the Work Ability Index Scale (WAI) to measure the transformation of the work ability of the study subjects is currently an effective method for evaluating the individual's work ability (39). The Work Ability Index Scale is a potential tool for identifying workers who are at risk due to imbalances in health, personal resources and job needs. The Chinese edition was revised by Ma et al. (40) and was verified to have good reliability and validity, and was suitable for relevant assessments in the field of occupational health in China, and the evaluation was relatively comprehensive. WAI scale includes seven aspects: WAI1 (self-evaluation of current work ability), WAI2 (current physical condition adapts to job requirements), WAI3 (current physical health), WAI4 (the impact of current injuries and illnesses on work), WAI5 (the number of days of absence due to illness in the past 12 months), WAI6 (prediction of one's ability to work in the next two years), and WAI7 (current mental health status). The total score of the scale is between 7 and 49 points, divided into four levels: (1) 7 to 27 points indicate poor working ability; (2) 28 to 36 points indicate that the working ability is medium and needs to be improved; (3) 37 to 43 points indicate good working ability; (4) 44 to 49 points indicate that the working ability is strong and can be well qualified for the current job (41).

### Structural equation model

**Figure 2:**
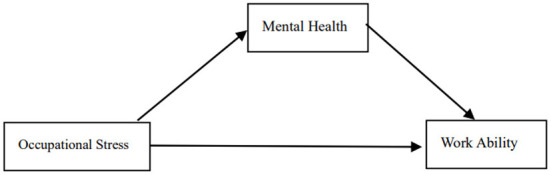
Hypothetical model.

Structural Equation Modeling (SEM) treats constructs that cannot be directly observed as latent variables (divided into exogenous and endogenous latent variables, the former as causes and the latter as effects), and directly observable as observed variables, using covariance matrices to identify, estimate, and verify the multi-cause and multi-effect relationship between latent variables and observed variables (42). On the basis of univariate analysis, the SEM of occupational stress, mental health and work ability of coal chemical workers in Xinjiang was further constructed. This study used Analytics of Moment Structures, AMOS statistical analysis software, according to the 7-step model construction process: theoretical setting (based on professional knowledge setting), model recognition (model freedom DF ≥ 0), survey data preparation, model simulation, model evaluation (generally considered to fit indicators to reach RMSEA < 0.08, GFI > 0.90, TLI > 0.90, CFI > 0.90 models are acceptable models), model correction and model interpretation to construct and validate relevant theoretical hypotheses in this study. Occupational stress, mental health, and ability to work were set as potential variables, and the indicators of the three scales are used as observation scalars. Establish structural equation models to analyze pathway relationships between occupational stress, mental health, and work ability. Based on the above literature, a hypothetical model (Figure 2) was constructed, and occupational stress directly affected mental health and ability to work. Mental health plays a mediating role in occupational stress and the ability to work.

### Quality control

All questionnaires were distributed to the coal chemical workers who were identified as the study subject and collected on-site. The questionnaire was completed anonymously within 15 min. At the initial stage of the survey, the trained investigators instructed the participants on how to complete the questionnaire, so that they could fully understand the significance of the research and ensure their active cooperation, which also encouraged the participants to truthfully and accurately complete each item of the questionnaire. During the investigation, two investigators conducted a comprehensive review of the completed questionnaires to ensure that any errors in the questionnaires were corrected promptly and that the missing items were added and completed in time, which also safeguarded the authenticity and effectiveness of the respondents' answers. During the data entry stage, this questionnaire was independently recorded and checked by two people, and relevant logical checks were carried out. After the data were inputted, statistical software was used to randomly check and recheck the database (i.e., according to the proportion of 20%) to ensure its accuracy.

### Statistical analysis

All data is entered into the Epi Data 3.1 database and analyzed using the SPSS 25.0 package. All metrological data were statistically described using?*x* ± *s*, two independent samples were compared to the *t*-test, and three and more sets of means were compared using one-way *ANOVA*. If there was a difference in the population, the *SNK-q* test was used for two-to-two comparisons. In the event that there was non-conformity with normality, non-parametric variance-based methods were used for the statistical analysis. The bias distribution is described in *M (P*_25_*, P*_75_*)*. Pearson correlation coefficients were used to analyze the correlation between occupational stress, mental health and work ability of coal chemical operators. Multi-factor analysis: The influence of general demographic factors, occupational stress and work burnout on work ability, and the interaction of occupational stress and mental disorders on work ability were analyzed using binary logistic regression. The AMOS 26.0 software was used to analyze the relationship between occupational stress, psychological disorder and work ability of coal chemical workers, and to fit the optimal structural equation model. The significance level is α = 0.05.

## Results

### Comparison of scores for occupational stress, mental health and work ability of coal chemical workers

In this study, 1,011 male workers (78.62% %) and female 275 (21.38 %). ERI scores were statistically significantly different between sex, age, length of service, education, shift work and monthly income (*P* < 0.05). SCL-90 scores were statistically different between different demographic characteristics (*P* < 0.05). The WAI scores varied significantly between age, length of service, education, marital status and monthly income (*P* < 0.05) (Table 1).

**Table 1:** Comparison of scores for occupational stress, mental health and work ability for different demographic characteristics.

**Variables**		***N***	**ERI score**	**SCL-90 score**	**WAI score**
Sex	Male	1,011	0.93 ± 0.25	104.00 (94.00, 132.00)	43.00 (39.00, 46.00)
	Female	275	0.85 ± 0.23	102.00 (94.00, 117.00)	43.00 (40.00, 47.00)
	*t /Z*		4.503	−2.047	−1.959
	*P-*value		<0.001	0.041	0.050
Age group, years	<30	398	0.93 ± 0.27	112.00 (94.00, 142.00)	42.00 (38.00, 45.00)
	30–45	416	0.93 ± 0.25	103.00 (93.00, 130.00)^a^	43.00 (39.00, 45.00)
	>45	427	0.88 ± 0.23^ab^	102.00 (92.00, 114.00)^ab^	44.00 (41.00, 46.00)^ab^
	*F/H*		7.124	48.837	49.764
	*P-*value		0.001	<0.001	<0.001
Working years	<5	161	0.92 ± 0.31	110.00 (96.00, 135.00)	43.00 (38.00, 45.00)
	5–20	481	0.95 ± 0.25	110.00 (96.00, 144.00)	42.00 (38.00, 45.00)
	>20	644	0.88 ± 0.23^d^	100.00 (92.00, 114.00)^cd^	44.00 (41.00, 46.00)^cd^
	*F/H*		9.757	60.603	53.870
	*P-*value		<0.001	<0.001	<0.001
Educational level	Junior high school or below	560	0.87 ± 0.23	99.00 (91.00, 111.00)	44.00 (41.00, 46.00)
	High school	583	0.93 ± 0.25^e^	110.00 (97.00, 141.00)^e^	43.00 (39.00, 46.00)^e^
	Associate's degree or above	143	0.99 ± 0.30^e^	115.00 (97.00, 155.00)^e^	40.00 (36.00, 44.00)^ef^
	*F/H*		15.689	105.626	40.078
	*P-*value		<0.001	<0.001	<0.001
Shift	Fixed day shift	352	0.85 ± 0.22	101.00 (92.00, 127.00)	46.00 (41.00, 49.00)
	Shift	934	0.94 ± 0.26	104.00(94.00, 128.00)	43.00(39.00, 45.00)
	*t /Z*		−6.243	−2.071	−11.026
	*P-*value		<0.001	0.038	<0.001
Marital status	Single	307	0.94 ± 0.27	111.00 (96.00, 142.00)	43.00 (38.00, 45.00)
	Married	979	0.91 ± 0.24	102.00 (92.00, 122.00)	43.00 (40.00, 46.00)
	*t/Z*		1.953	−4.390	−3.029
	*P-*value		0.051	<0.001	0.002
Monthly income	<3,000	324	0.87 ± 0.24	101.00 (92.00, 116.00)	44.00 (40.00, 47.00)
	3,000–5,000	847	0.93 ± 0.26^g^	105.00 (94.00, 135.00)^g^	43.00 (39.00, 45.00)^g^
	>5,000	115	0.90 ± 0.23	103.00 (95.00, 124.00)	44.00 (41.00, 46.00)^h^
	*F/H*		7.307	11.347	20.969
	*P-*value		0.001	0.003	<0.001

Compared with the age group < 30 years old, ^a^P < 0.05; Compared with the 30–45-year-old group, ^b^P < 0.05; Compared with the working years < 5 years, ^c^P < 0.05; Compared with the working years 5–20 years group, ^d^P < 0.05; Compared with an education level of junior high school and below, ^e^P < 0.05; Compared with an education level of high school, ^f^P < 0.05; Compared with the group of monthly income < 3,000 yuan, ^g^P < 0.05; Compared with the group of monthly income 3,000–5,000 yuan, ^h^P < 0.05.

### Comparison of work abilities of different occupational stress groups

A comparison of the work ability levels of coal chemical workers at different occupational stress levels showed that there were 860 people in ERI ≤ 1 (low-stress group) and 426 people in ERI >1 (high-stress group). The incidence of poor working ability in the high occupational stress group of coal chemical workers was higher than that in the low occupational stress group (χ^2^ = 234.622, *P* < 0.001) (Table 2).

**Table 2:** Comparison of work abilities of different occupational stress groups.

**Grouping**	***N***	**Ability to work level**				**χ^2^**	***p*-value**
		**Poor**	**Medium**	**Good**	**Excellent**		
ERI ≤ 1	860	4 (0.47%)	55 (6.40%)	271 (31.51%)	530 (61.63%)	234.622	<0.001
ERI > 1	426	7 (1.64%)	107 (25.12%)	233 (54.69%)	79 (18.54%)		

### Comparison of working abilities of different groups of mental disorders

The results of comparing the work ability level of coal chemical workers in different mental disorder groups showed that the incidence of poor working ability in the group of coal chemical workers with mental disorders was higher than that in the group without mental disorders (χ^2^ = 225.806, *P* < 0.001) (Table 3).

**Table 3:** Comparison of work abilities of different groups with mental disorders.

**Mental disorders**	***N***	**Ability to work level**				**χ^2^**	***p*-value**
		**Poor**	**Medium**	**Good**	**Excellent**		
No	996	3 (0.30%)	64 (6.43%)	370 (37.15%)	559 (56.12%)	225.806	<0.001
Yes	290	8 (2.76%)	98 (33.79%)	134 (46.21%)	50 (17.24%)		

### Analysis of occupational stress, mental disorders and work ability of coal chemical workers

Pearson correlation analysis method was used to analyze the correlation between occupational stress, mental disorder and work ability of coal chemical workers. The results showed that work ability and its dimensions were inversely correlated with external effort, occupational stress and mental disorders (*P* < 0.001). The ability to work and its dimensions were positively correlated with reward (*P* < 0.05) (Table 4).

**Table 4:** Correlation analysis of occupational stress, mental disorders and work ability of coal chemical workers.

**Grouping**	**External effort**	**Reward**	**Intrinsic input**	**Occupational stress**	**Mental disorders**
WAI1	−0.165**	0.157**	−0.006	−0.236**	−0.243**
WAI2	−0.166**	0.140**	−0.070*	−0.227**	−0.264**
WAI3	−0.190**	0.097*	−0.198**	−0.195**	−0.484**
WAI4	−0.168**	0.132**	−0.082*	−0.220**	−0.192**
WAI5	−0.072*	0.079*	0.004	−0.101**	−0.092**
WAI6	−0.125**	0.117**	−0.035	−0.177**	−0.159**
WAI7	−0.127**	0.162**	−0.011	−0.200**	−0.232**
Work ability	−0.270**	0.221**	−0.123**	−0.352**	−0.460**

*P < 0.05, **P < 0.001.

### Effects of the interaction of occupational stress and mental disorders on work ability

Taking low occupational stress ^*^ no mental disorders as a control, the two-by-two interaction between different occupational stress and mental disorders groups is included in the equation. The results showed that the interaction of occupational stress and mental disorders had a significant effect on sleep quality (*p* < 0.001). Workers with high occupational stress^*^ mental disorders (OR = 10.666, 95% CI: 6.443–17.658) are 10.666 times more likely to be at risk for developing poor work ability than low occupational stress^*^ no mental disorders (Table 5).

**Table 5:** Effects of the interaction of occupational stress and mental disorders on work ability.

**Variables**	**B**	**S.E**.	**Wald**	**P-value**	**OR**	**95%CI**
Constant	−3.229	0.193	280.789	<0.001	0.040	-
High stress* no mental disorder	1.069	0.280	14.578	<0.001	2.913	1.683–5.044
Low stress* mental disorder	1.236	0.336	13.542	<0.001	3.443	1.782–6.652
High stress* mental disorder	2.367	0.257	84.691	<0.001	10.666	6.443–17.658

### Structural equation model of occupational stress-mental health-work ability

**Figure 3:**
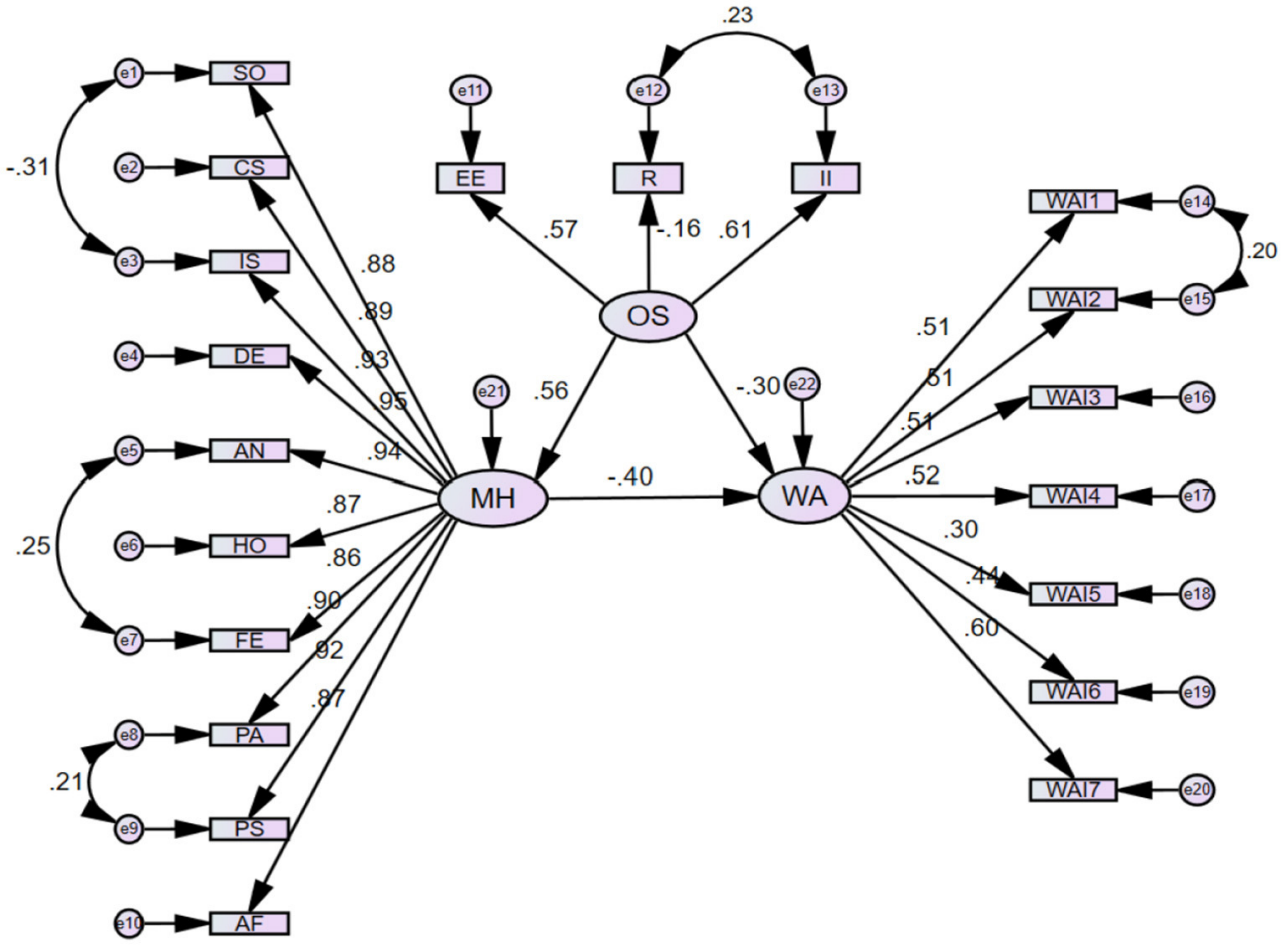
Structural equation modeling results. The structural model has an adequate fit for the data. All the coefficients in this figure are standardized and significant at the 0.05 level. OS, Occupational Stress; II, Intrinsic Input; R, Reward; EE, External Effort; MH, Mental Health; SO, Somatization; CS, Compulsive Symptoms; IS, Interpersonal Sensitivity; DE, Depression; AN, Anxiety; HO, Hostility; FE, Fear; PA, Paranoia; PS, Psychosis; AF, Another Factors; WA, Work Ability.

Using AMOS 26.0 software for maximum likelihood estimation testing, through the continuous modification of the model, the following model is selected according to the principles of accuracy and simplicity. Throughout the sample, the mediation effect model fit for mental health was satisfactory in the process of working stress to work capacity (Figure 3). The models fit well (RMSEA = 0.066, AGFI = 0.902, GFI = 0.924) and the differences were statistically significant (*P* < 0.001). The results of structural equation modeling indicate that occupational stress has a direct negative effect (r = −0.30, *P* = 0.002) on work ability and also an indirect effect on mental health. The intermediate effect was 0.56 × −0.40 = −0.224, accounting for 42.75% of the total effect.

## Discussion

The results of this study showed that the working ability score of Xinjiang coal chemical workers was 43.00 (40.00, 46.00), and 1,055 people with excellent and good working ability, accounting for 82.04%, of which were women. Studies have shown that women score relatively higher on work ability than men, unlike Fischer and Martinez (43) findings. This may be related to the difficulty and severity of the tasks assigned to the enterprise, and women are mostly in positions with relatively easy workloads. The higher the age and length of service of coal chemical workers, the higher the work ability index scores, which is the same as the results of the Umanath and Marsh (44) study. It may be that the higher the age and working age, the richer the accumulated work experience, and the greater the familiarity with the work business, the less pressure. However, studies have also shown that the ability to work gradually declines with age (45). Unmarried coal chemical workers score lower than married people, indicating that a stable marital relationship is beneficial to work ability. Studies have shown that married working women have a negative relationship with depression and anxiety, which may also account for the higher work capacity of married women (46, 47). Coal chemical workers who work shifts score lower work ability than workers on a fixed day shift. Li et al. (48) studies of oil workers and Merchaoui et al. (49) studies of nurses both showed that shifts reduced workers' ability to work. Frequent and irregular shift work may have a negative impact on work capacity. It is suggested that enterprises should arrange the work shift system reasonably to improve the work ability and life of workers (50).

This study aimed to explore the relationship between occupational stress, psychological disorders and work ability among coal chemical workers in Xinjiang. The results showed that coal chemical workers in the low-level occupational stress group had higher work ability than in the high-level occupational stress group. Studies have shown that prolonged occupational stress depletes workers' motivation, leading to a decrease in work capacity and lowering the productivity of the company (51, 52). Occupational stress has become a cause of health damage, absenteeism, and workplace accidents (53). Compared with the group without mental disorders, the incidence of poor working ability and poor coal chemical workers in the mental disorder group was higher. Studies have shown that psychological problems not only have a negative effect on individual emotions and interpersonal relationships but also have a negative effect on work-related fatigue and increased sick leave (54). Other studies have found that employees' mental health affects their job performance, which is reflected in an increased risk of making mistakes and a decline in job performance (55).

The correlation analysis between occupational stress, mental disorders and work ability showed that work ability and its various dimensions were negatively correlated with external effort, occupational stress and mental disorder, and positively correlated with reward. Numerous studies have also shown that occupational stress and mental disorders are negatively correlated with the ability to work (21, 56). The higher the return, the better able to work. Therefore, enterprises should take corresponding measures to reward employees with good work performance and increase the remuneration of workers to improve the working ability of coal chemical workers. Through multiple linear regression analysis of the factors affecting work ability. The results showed that in addition to the impact of factors, such as age and shift, the working ability of coal chemical workers was also affected by occupational stress and mental disorders. The results showed that the interaction between occupational stress and mental disorder had a significant impact on work ability. High occupational stress^*^ mental disorders (OR = 10.666, 95% CI: 6.443–17.658) are 10.666 times more likely to develop poor work ability than low occupational stress^*^ no mental disorders. Research by Lindegård et al. (57) and Carlisle and Parker (58) also suggested that occupational stress and mental disorder are risk factors affecting the ability to work.

This study examined the potential mediating pathways of mental disorders in occupational stress and work ability in coal chemical workers. SEM is a model based on a regression model that offers advantages over traditional methods such as multiple regression, factor analysis, and covariance analysis (59). This study also confirmed the intrinsic relationship between variables by building structural equation models. One finding confirmed in our model was that the mediating pathway through mental health has an important regulatory role in the indirect effect of occupational stress on the ability to work, which explains about 42.75% of the effects. Through the model fitting path, it was found that occupational stress and mental health conditions have a direct negative effect on work ability. Occupational stress had a direct positive effect on mental health conditions, similar to the results of the Moreno Fortes et al. (60) study. Occupational stress also had an indirect negative effect on the ability to work through mental health conditions. Higher levels of occupational stress were associated with poorer mental health levels. Relevant studies have also shown that in groups such as firefighters (61), police officers (62), medical staff (63), and workers (64), the level of occupational stress affected the mental health status to different degrees. Theleritis's research suggests that firefighters are continuously exposed to intense stressful situations and traumatic events and are at high risk for post-traumatic stress disorder (PTSD). Coping mechanisms and behaviors have been studied as factors contributing to PTSD (65). In addition, other studies (66) have shown that during the extraordinary period of the COVID-19 pandemic, anesthesiologists, community workers, and other groups also experienced a high level of occupational stress, which led to anxiety, depression, and the onset of other symptoms. It shows that the occupational stress of the coal chemical industry can directly act on work ability, and can also indirectly act on work ability through mental health status as an intermediary variable. Future research could continue to explore the effects of PTSD, and depression on workers' ability to work.

## Conclusion

The interaction between occupational stress and mental disorders has a significant impact on work ability stress can act directly on work ability, or it can act indirectly on work ability through mental health conditions as a mediating variable. Relevant departments and work units can adopt corresponding policies and measures to reasonably arrange work tasks and shift systems, thereby reducing the level of occupational stress of coal chemical workers and improving their work ability. At the same time, pay attention to the psychological conditions of coal chemical workers, through psychological counseling and counseling, increase rest and entertainment methods, alleviate workers' nervousness, and promote their physical and mental health development, thereby comprehensively improving their health level and quality of life of workers.

### Strengths and limitations

This study investigated the relationship between occupational stress, psychological disorders, and work ability. The structural relationship between mental health, occupational stress, and work ability was explored in depth, which has been studied in a few studies. Even though this research was a cross-sectional study, a randomized sampling method was employed. Valid and reliable questionnaires (validated in Chinese) were used, which minimized bias. However, this study also had some limitations. First, the causal relationship cannot be determined for the current investigation of this study; future studies should further select methods such as cohort studies. Second, some of the findings in this paper are different from the results of previous scholars, which may be related to the selection of samples, especially the proportion of male workers and female workers. The inclusion of future study subjects should be balanced to make them more representative. This study used the SCL-90 for the assessment of mental disorders, and the participants were not actually examined by a certified psychiatrist, so there may be some bias or other errors in accurately reporting mental status, psychological characteristics, etc. Finally, due to a large number of coal chemical enterprises in Xinjiang, the research objects have not been fully covered, and the results of this study cannot represent the mental health and working ability of all coal chemical workers in Xinjiang, so in the future, the research should investigate the workers of various coal chemical enterprises, minimize the deviation, and verify the conclusions of this study.

## Data availability statement

The original contributions presented in the study are included in the article/supplementary material, further inquiries can be directed to the corresponding author.

## Ethics statement

The research proposal was approved by the Ethics Committee of Xinjiang Medical University. Prior to the start of the survey, all respondents voluntarily provided written informed consent forms. The patients/participants provided their written informed consent to participate in this study. Written informed consent was obtained from the individual(s) for the publication of any potentially identifiable images or data included in this article.

## Author contributions

XY, JY, XG, and FL conceived and designed the study, contributed to the acquisition, analysis, interpretation of data, were involved in drafting the manuscript, and revising it for important intellectual content. All authors contributed substantially to the work presented in this paper, reviewed, and approved the final manuscript.

## Funding

This work was funded by the National Natural Science Foundation of China (Grant Numbers: 81660533 and 82060590) and the Key Discipline of the 13th 5-Year Plan in Xinjiang Uygur Autonomous Region-Public Health and Preventive Medicine.

## Conflict of interest

The authors declare that the research was conducted in the absence of any commercial or financial relationships that could be construed as a potential conflict of interest.

## Publisher's note

All claims expressed in this article are solely those of the authors and do not necessarily represent those of their affiliated organizations, or those of the publisher, the editors and the reviewers. Any product that may be evaluated in this article, or claim that may be made by its manufacturer, is not guaranteed or endorsed by the publisher.
